# Circumpolar Diversity and Geographic Differentiation of mtDNA in the Critically Endangered Antarctic Blue Whale (*Balaenoptera musculus intermedia*)

**DOI:** 10.1371/journal.pone.0032579

**Published:** 2012-03-07

**Authors:** Angela L. Sremba, Brittany Hancock-Hanser, Trevor A. Branch, Rick L. LeDuc, C. Scott Baker

**Affiliations:** 1 Department of Fisheries and Wildlife and Marine Mammal Institute, Oregon State University, Hatfield Marine Science Center, Newport, Oregon, United States of America; 2 Protected Resources Division, Southwest Fisheries Science Center, National Marine Fisheries Service, National Oceanic and Atmospheric Administration, La Jolla, California, United States of America; 3 School of Aquatic and Fishery Sciences, University of Washington, Seattle, Washington, United States of America; Barnard College, Columbia University, United States of America

## Abstract

The Antarctic blue whale (*Balaenoptera musculus intermedia*) was hunted to near extinction between 1904 and 1972, declining from an estimated initial abundance of more than 250,000 to fewer than 400. Here, we describe mtDNA control region diversity and geographic differentiation in the surviving population of the Antarctic blue whale, using 218 biopsy samples collected under the auspices of the International Whaling Commission (IWC) during research cruises from 1990–2009. Microsatellite genotypes and mtDNA sequences identified 166 individuals among the 218 samples and documented movement of a small number of individuals, including a female that traveled at least 6,650 km or 131° longitude over four years. mtDNA sequences from the 166 individuals were aligned with published sequences from 17 additional individuals, resolving 52 unique haplotypes from a consensus length of 410 bp. From this minimum census, a rarefaction analysis predicted that only 72 haplotypes (95% CL, 64, 86) have survived in the contemporary population of Antarctic blue whales. However, haplotype diversity was relatively high (0.968±0.004), perhaps as a result of the longevity of blue whales and the relatively recent timing of the bottleneck. Despite the potential for circumpolar dispersal, we found significant differentiation in mtDNA diversity (F_ST_ = 0.032, p<0.005) and microsatellite alleles (F_ST_ = 0.005, p<0.05) among the six Antarctic Areas historically used by the IWC for management of blue whales.

## Introduction

The Antarctic blue whale was among the most valuable of the exploited great whales [1]. From the outset of modern whaling in 1904 to the end of Soviet illegal whaling in 1972, more than 345,000 Antarctic blue whales were killed [2]. This resulted in a decline from an estimated pre-exploitation abundance of between 235,000 and 307,000 to less than 400 (95% CL, 150–840) individuals in 1972 [3], [4]. By 1998, the population was estimated to have increased to 2,280 (95% CL, 1,160–4,500), based on sighting surveys conducted under the auspices of the International Whaling Commission (IWC) during the International Decade of Cetacean Research and Southern Ocean Whale and Ecosystem research cruises (IDCR/SOWER) [5].

**Figure 1:**
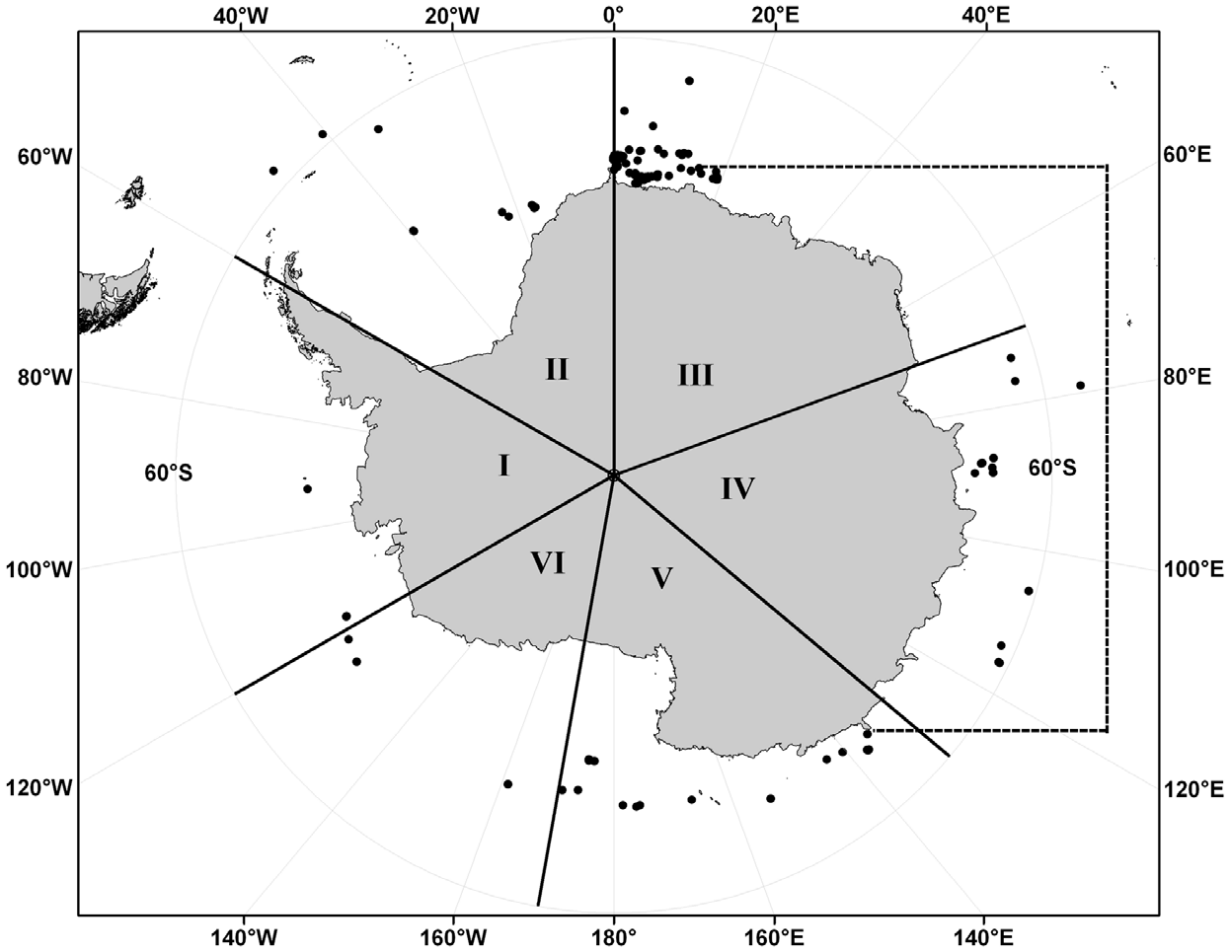
Locations of biopsy samples collected from Antarctic blue whales during IDCR/SOWER cruises from 1990 and 2009. Solid lines demarcate IWC management Areas (I–VI). Dashed line depicts inferred movement of an Antarctic blue whale between Areas over an elapsed time of 4 years based on genotyped recapture locations (Z-51452).

Historically, the IWC considered that Antarctic blue whales formed six ‘stocks,’ or demographically isolated biological populations, within which internal dynamics (births and deaths) are more important than external dynamics (immigration and emigration) [6]. These stock divisions were based on the apparent concentrations and discontinuities in the distribution of blue whale catches during early 20^th^ century commercial whaling in the Southern Ocean [6], [7]. The approximate boundaries of these stocks are recognized today by the IWC as the Antarctic management Areas I to VI (Figure 1) [6]. However, the only direct evidence of population structure in the Antarctic blue whale has come from the ‘*Discovery*’ marking program during the commercial whaling era. ‘*Discovery*’ marks were steel darts stamped with a unique serial number and fired into the muscle of the whale with a modified shotgun [8]. The mark was recovered if the whale was killed and flensed. From ‘*Discovery*’ marking and recoveries, the longest range of inferred longitudinal movement was over 180°or 6,250 km (marking location: 65.5°S, 80.8°W; recapture location: 57.9°S, 87.8°E). This marking and recovery was recorded over an elapsed time of four years [9].

The effects of exploitation and the influence of population structure on surviving genetic diversity of Antarctic blue whales remain poorly described. A major obstacle to a comprehensive survey of diversity and differentiation in this circumpolar population has been access to biological samples. Unlike southern right whales (*Eubalaena australis*) and humpback whales (*Megaptera novaeangliae*), which are relatively accessible to sampling in coastal waters during the winter breeding season [10], [11], the breeding grounds of Antarctic blue whales are poorly defined and sampling has been restricted to the feeding grounds of the Southern Ocean. Given the rarity of Antarctic blue whales and their preference for feeding habitat south of the Antarctic convergence (54°S–55°S), it has required 14 years and three circumpolar surveys by the IDCR/SOWER program to collect biopsy samples from all six Antarctic management Areas (Table 1). Another complication in describing the diversity of the Antarctic blue whale is the potential for some overlap in distribution or misidentification with a second subspecies in the Southern Hemisphere, the pygmy blue whale (*B. m. brevicauda*). The pygmy blue whale was first differentiated in catch records based on its smaller size and more northerly distribution during the summer feeding season [12]. During the austral summer, the Antarctic blue whale congregates in the Southern Ocean near the pack ice south of 55°S, while the pygmy blue whale is found primarily north of 54°S (between 40°S and 54°S) [13]. Mixture models based on an extensive review of catch records have estimated that only 0.1% of blue whales south of 52°S were pygmy blue whales, and 0.5% of blue whales north of 52°S were Antarctic blue whales [14]. In other words, the probability of misclassifying the two subspecies by location of catches during the austral summer was low.

**Table 1:** Number of biopsy samples of Antarctic blue whales collected between 1990 and 2009 (n = 218) on IDCR/SOWER cruises.

Area	1990	1991	1992	1993	1994	1995	1996	1997	1998	1999	2000	2001	2002	2003	2004	2005	2006	2007	2008	2009	Total n
I	1				0						0	4									5
II								4	6												11
III				2		0										4	36	85			127
IV						2				10						1			0	9	22
V			0										16	5	14						35
VI		0					0					18									18
Total	1	0	0	2	0	2	0	4	6	10	0	22	16	5	14	5	36	85	0	9	218

Frequency of samples collected in each Area and year are listed as well as within Area (I–VI) and yearly totals. ‘Year’ is referenced to the end-date of the annual surveys, e.g., 1990 refers to the 1989/1990 austral summer. Zeros indicate Areas that were surveyed but in which no biopsy samples were collected that year.

The only previous study describing genetic diversity and differentiation of the Antarctic blue whale focused primarily on discriminating this subspecies from two other populations assumed to represent pygmy blue whales: one in the South-east Pacific and one in the Indian Ocean [15]. For this comparison, LeDuc et al. [15] had access to samples collected during IDCR/SOWER cruises prior to 2002 and to samples collected by the Japanese Whale Research Program under Special Permit in the Antarctic (JARPA). Together the IDCR/SOWER and JARPA samples represented 46 Antarctic blue whales, as classified by sampling location south of the Antarctic convergence, as well as 36 pygmy blue whales from the Indian Ocean and 28 from the South-east Pacific. LeDuc et al. [15] found little evidence of taxonomic distinctiveness between the two subspecies, based on a phylogenetic reconstruction of mtDNA sequences, but did find highly significant differentiation between the three Southern Hemisphere populations based on mtDNA haplotype frequencies and a high probability of population assignment using seven microsatellite loci [15]. From this, LeDuc et al. [15] suggested that the subspecific taxonomy should be re-examined, but concluded that the Antarctic blue whale represented a distinct population segment [16] and that classification of Antarctic blue whales based on location was accurate.

Although LeDuc et al. [15] did not consider their sample size to be sufficient for an analysis of geographic structure in the Antarctic blue whale, they were able to report the first estimate of mtDNA diversity in the population and a minimum census of 26 haplotypes among 46 individuals. From this minimum census, Branch and Jackson [17] used a rarefaction approach to predict that a total of only 51 haplotypes (i.e., haplotype richness) survive in the contemporary population. This prediction is of interest because haplotype richness can be used in population dynamic modeling to inform a lower boundary on the minimum number of whales (*N_min_*) that survived the exploitation bottleneck caused by commercial whaling [18].

Here we update the previous estimate of mtDNA diversity and the minimum number of surviving maternal lineages of the Antarctic blue whale by analysis of 218 samples collected on IDCR/SOWER cruises from 1990–2009, including those used by LeDuc et al. [15]. From this circumpolar sample, we also describe the distribution of mtDNA haplotype diversity within the Southern Ocean and test for geographic structure for both mtDNA and microsatellite data based on the *a priori* IWC management Areas. Through the identification of replicate samples using microsatellite genotypes (i.e., genotyped recaptures), we document the individual movement of Antarctic blue whales, providing the first evidence of large-scale movement since ‘*Discovery*’ marking ended nearly 50 years ago.

## Methods

### Sample collection and ethical statement

Biopsy samples of blue whales were collected in international waters during IDCR/SOWER cruises aboard vessels operated by the Government of Japan, according to Japanese domestic laws. All cruises included oversight by international scientists appointed by the Secretariat of the IWC and methods for the collection of biopsy samples were consistent with those approved for use with large whales by the Animal Ethics Committee of the University of Auckland (e.g., AEC/02/2002/R9 and AEC/02/2005/R334 to C.S. Baker). Samples were transferred from Japan for archiving at the Southwest Fisheries Science Center (SWFSC) of the US National Marine Fisheries Service under CITES permits US774223, 08US774223/9, and 09US774223/9 and US Marine Mammal Protection Act permits 1026/689424, 774–1437, 774–1714, and 14097.

The IDCR/SOWER cruises follow systematic track lines for the primary purpose of estimating the circumpolar abundance of baleen whales in the Southern Ocean by distance sampling, but departed from track lines for the secondary purpose of collecting biopsy samples. During the IDCR programs, surveys were usually conducted in one Area each austral summer with the intent of completing a circumpolar survey in about six years. Three circumpolar surveys (CP) were completed from 1978 to 2004 with additional Area specific surveys during the SOWER program from 2005–2009 (Table 1; for details of survey design see Ensor [19] and Branch and Butterworth [20]). A total of 218 samples collected from 1990 to 2009 were classified as ‘Antarctic blue whale’, based on location of collection, south of the Antarctic convergence (54°–55°S) [9], [13], [14]. Sampling locations spanned all six Antarctic management Areas (Figure 1; Table 1) and differences in sample sizes corresponded roughly to estimated abundance from the most recent circumpolar survey (CPIII), except for Area III where a series of surveys were concentrated after 2003/04 as a part of the SOWER program [21], [22].

After transfer of the biopsy samples to the SWFSC, DNA was extracted following a variety of methods, including lithium chloride extraction [23], standard phenol chloroform extraction [24], sodium chloride protein precipitation [25], and silica-based filter purifications (DNeasy kit, Qiagen, Valencia, CA, USA). Access to DNA from the IDCR/SOWER samples was granted by a proposal to the Scientific Committee of the IWC. A subset of these samples (n = 29) was used in the previous genetic analysis of Antarctic blue whales by LeDuc et al. [15] and made available here for inclusion in all laboratory analyses. Samples collected by the Japanese Whale Research Program under Special Permit in the Antarctic (JARPA) (n = 17) were also included in LeDuc et al. [15] but were not requested in this loan; however, published sequences, sex and genotypes were available in Supplementary Material provided by LeDuc et al. [15].

### Sex identification and microsatellite genotyping

To identify replicates within the dataset, samples were genotyped at up to 16 microsatellite loci (Table 2). All microsatellites were amplified individually (i.e. no PCR multiplexes) in 10 µL volumes using 1× buffer (Invitrogen), 2.5 mM MgCl_2_, BSA (Bovine Serum Albumin), 0.4 µM of labeled primers, 0.1 mM dNTPs, Platinum *Taq* DNA Polymerase (Invitrogen) and 1 µL of template DNA. Reaction conditions used the following thermocycle profile: denaturing for 3 minutes at 94°C, 30 cycles of denaturing at 94°C for 30 seconds, annealing at 55°C for 45 seconds; and extension at 72°C for 60 seconds, followed by a final extension step of 72°C for 10–30 minutes depending on the locus. Amplicons were co-loaded for genotyping in 4 sets of up to 5 loci. For each sample, 2 µl of co-load in addition to size standard GS500 LIZ (Applied Biosystems, Foster City, CA) and HiDi Formamide (Applied Biosystems, Foster City, CA) were heated to 95°C for 5 minutes and genotyped on an ABI 3730xl (Applied Biosystems, Foster City, CA). Automated calling of alleles by GENEMAPPER v4.0 (Applied Biosystems, Foster City, CA) was confirmed by a visual inspection for each sample. Microsatellite genotypes were reviewed using the program CERVUS v3.0 [26] to identify likely replicates. The program Micro-checker was used to check for genotype error and null alleles [27]. Deviations from Hardy Weinberg Equilibrium were checked for each locus in Genepop v4.0.10 [28], [29].

**Table 2:** Summary of microsatellite loci used to identify likely replicate samples in Antarctic blue whale samples, with test for Hardy Weinberg equilibrium (HWE) and test of differentiation.

Locus	n (samples)	k	p(ID)	n (individuals)	HWE(p-value)	F_ST_	(p)	Ref.
GT575	198	8	0.164	145	0.848	<0.001	0.195	[64]
rw4–10	193	17	0.026	134	0.218	<0.001	0.876	[65]
Ev37*	188	11	0.228	137	0.308	0.036	0.204	[66]
Ev96	201	10	0.064	148	0.015	0.017	0.089	[66]
464.465	196	6	0.090	145	0.112	0.018	0.063	[67]
GT23*	190	10	0.072	142	0.031	0.013	0.040	[64]
Ev104	178	10	0.123	140	0.002**	0.005	0.433	[66]
Overall(7 loci)						0.011	0.032	
Ev1	182	19	0.020	122	0.115	0.004	0.078	[66]
Ev94	161	3	0.686	124	0.123	<0.001	0.407	[66]
GATA417*	150	15	0.029	116	0.000**	<0.001	0.029	[68]
GATA28*	147	9	0.046	114	0.882	<0.001	0.813	[68]
GT211	169	10	0.077	127	0.031	<0.001	0.739	[64]
rw31	162	3	0.789	127	1.000	<0.001	0.688	[65]
rw48	152	13	0.109	116	0.677	0.025	0.226	[65]
Ev14	154	10	0.061	116	0.040	0.015	0.152	[66]
Ev21	148	9	0.061	111	0.002**	<0.001	0.605	[66]
Overall (16 loci)			7.9×10^−17^		<0.001	0.005	0.032	

Locus name, number of samples genotyped, number of identified alleles (k), probability of identity (p(ID)), number of individuals genotyped, probability of Hardy Weinberg equilibrium (** notes loci out of HWE after a sequential Bonferonni correction), F_ST_ and significance (p-value) and original reference (Ref.) are listed for each loci. Overall F_ST_ and p values for 7 and 16 loci are listed. Asterisks (*) denote microsatellite loci included in LeDuc et al. [15].

Sex was identified by multiplex PCR using primers P1–5EZ and P2–3EZ to amplify a 443–445 bp region on the X chromosome [30] and primers Y53-3C and Y53-3D to amplify a 224 bp region on the Y chromosome [31]. Reaction conditions and thermocycle profiles were the same as for microsatellite loci except denaturing at 94°C for 45 seconds and annealing temperature at 60°C for 45 seconds.

### mtDNA amplification and sequencing

An approximately 800 bp fragment of the mtDNA control region was amplified with the forward primer M13Dlp1.5 (e.g. Dalebout et al. [32] 5′ TGTAAAACGACGGCCAGTTC ACCCAAAGC TGRARTTCTA 3′) and reverse primer Dlp8G (5′ GGAGTACTATG TCCTGTAACCA 3′), under standard conditions [33]. In preparation for sequencing, excess dNTPs and primers were removed from amplified mtDNA control region products using shrimp alkaline phosphotase and exonuclease I (SAPEX - Amersham Biosciences), and a dye termination sequencing reaction was carried out using BigDye Dye Terminator Chemistry v3.1 (Applied Biosystems Inc.), following the manufacturer's protocol. Unincorporated bases and dyes were removed using CleanSEQ (Beckman Coulter Genomics) and the product was run on an ABI 3730xl. Chromatograms were reviewed for quality control using ABI quality control scores in Sequencher v4.6 (GeneCodes). Any sequences with Phred scores <30 were repeated [34]. Sequences were trimmed to a consensus length of 560 bp.

Although IDCR/SOWER samples were sequenced to 560 bp in length, haplotypes were defined based on substitutions within the first 410 bp for comparison to published sequences from other Southern Hemisphere populations in the Indian and South-east Pacific Oceans [15]. Unique haplotypes that were not found in the Southern Hemisphere database were reverse sequenced from an independent amplification for verification of variable sites. Previously unreported haplotypes were named according to the SWFSC lab code of the first sample found to have that haplotype. Estimates of haplotype (*h*) and nucleotide (π) diversity as well as Tajima's D and Fu's F test of neutrality were calculated in Arlequin v3.1 [35].

### Estimating haplotype richness

A rarefaction analysis and discovery curve were used to estimate the total number of surviving haplotypes (i.e., haplotype richness) in the contemporary Antarctic blue whale population following the methods of Jackson et al. [18]. Unlike standard rarefaction analysis, this approach provides an estimate of the total number of haplotypes in the contemporary Antarctic blue whale population by including both the uncertainty in the sampling of haplotypes and the uncertainty in the estimate of population size. In brief, a Clench function is fit to the discovery curve (i.e., cumulative number of haplotypes plotted against number of samples) using the formula:
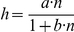
where *h* is the predicted haplotype richness for a sample of size *n*, and *a* and *b* are parameters to be estimated. Once estimates are obtained for *a* and *b*, the sample size *n* is replaced with the total population estimate *N* to estimate the total number of haplotypes in the population itself.

To incorporate the uncertainty associated with the sampling process in this population, the samples in this study were randomly reordered 10,000 times to mimic different possible sampling orders. The range in number of unique haplotypes at the varying sample sizes was recorded and this information was used with the Clench function to calculate 10,000 estimates for parameters *a* and *b.* To incorporate uncertainty around estimates of total population size in this species, the best available estimate of population abundance of 2,280 (95% CL, 1,160–4,500) [5] for the year 1998 was approximated by a lognormal distribution with mean = ln(2,280) and SD = 0.345. Distributions of the total number of haplotypes present in the Antarctic blue whale population were then generated using 10,000 random draws taken from the estimates of parameters *a* and *b,* and the estimates of the population size drawn from the lognormal distribution.

### Phylogenetic reconstruction and geographic differentiation

The phylogenetic relationship of mtDNA haplotypes from the Antarctic blue whale and other Southern Hemisphere blue whale populations was reconstructed in PAUP* v4 [36] and BEAST v1.6.1 [37]. The model parameters used in these programs were obtained through jModeltest [38], [39] and MrModelTest v2.3 [40], respectively. After removal of replicate samples identified by microsatellite genotyping, geographic differentiation in mtDNA haplotypes was measured between the management Areas I–VI using F_ST_ and Φ_st_ calculated in Arlequin v3.1 [35]. We also tested for mtDNA differentiation within males and females where sex information was available. One individual sampled in more than one Area was included in both Areas for the tests of genetic differentiation. Significance of genetic differentiation was tested using 5,000 random permutations of the data matrix, as well as the test of differentiation (modified exact test) in Arlequin v3.1 [35]. Differentiation in microsatellite alleles between the management Areas I–VI was measured using F_ST_ with statistical significance based on 5,000 iterations of an exact test in Genepop v4.0.10 [29], [41]. As a Bonferonni correction is considered overly conservative for assessing population units of endangered species, we report p-values for the test of differentiation with, and without, the sequential Bonferonni correction. To explore the possibility of cryptic structure not accounted for by management Areas, we also analyzed microsatellite genotypes using STRUCTURE v2.3.1 [42]. STRUCTURE analyses were run with k = 1–7 populations for six iterations with 1,000,000 repetitions after a burn-in period of 100,000. The Δk method was used to select the most likely value of k [43].

## Results

### Individual identification and movement

A total of 218 samples were available from the IDCR/SOWER surveys, representing all six Antarctic management Areas. Of these, 215 samples provided high quality sequences for 560 bp of the mtDNA control region. The remaining three samples did not amplify for mtDNA but could be included in our analysis of mtDNA diversity using information from the Supplementary Materials of LeDuc et al. [15]. Although variation in quality and quantity of DNA resulted in some other failures in amplification of microsatellite loci and sex [15], we were able to genotype the 215 samples at an average of 12.9 of the 16 microsatellite loci. Based on matching of microsatellite genotypes, mtDNA sequences and sex, we identified 52 likely replicates among the 215 samples. Of these, 38 replicates of 31 individuals were collected during the same encounter and provided no information on individual movement. Another 11 replicates of seven individuals provided information on movement across a few days, and three replicates of two individuals provide evidence of movement across one to four years (Table 3, Figure 1). These nine genotype ‘recapture’ events were established with high confidence, based on their estimated probability of identity (p(ID)). One individual (Z-51452), a female, was first sampled in Area V in 2002 and recaptured in Area III in 2006 ((p(ID) = 6.80×10^−18^) a longitudinal displacement of ∼131°. Another individual (Z-62489), a male, was first sampled in Area III in 2002 and recaptured in the same Area in 2007 (p(ID) = 3.07×10^−12^) (Figure 1). After removal of all replicate samples, the final dataset included 166 individuals, one of which was sampled in two of the IWC management Areas.

**Table 3:** Location and dates of recaptures of individual Antarctic blue whales identified from genotype matching.

Capture occasion	LAB ID code	Area	Date	Lat°	Long°	Matching loci	Distance km	p(ID)
**Between Years**								
**Capture**	Z-51452	V	6-Jan-2002	−64.32	137.27			
Recapture	Z-62484	III	26-Jan-2006	−69.38	5.43	14	6,650	6.80×10^−18^
**Capture**	Z-62489	III	29-Jan-2006	−67.32	12.32			
Recapture	Z-72957	III	7-Feb-2007	−69.60	5.83	13	400	3.07×10^−12^
	Z-72959	III	7-Feb-2007	−69.60	5.83	15		1.20×10^−15^
**Within Year**								
**Capture**	Z-62501	III	9-Feb-2006	−68.48	18.55			
Recapture	Z-62508	III	13-Feb-2006	−68.42	14.23	16	200	1.50×10^−21^
**Capture**	Z-72908	III	7-Jan-2007	−68.70	0.45			
Recapture	Z-72903	III	8-Jan-2007	−67.58	2.75	16	125	1.00×10^−19^
	Z-72904	III	8-Jan-2007	−67.58	2.75	16		1.00×10^−19^
**Capture**	Z-72906	III	8-Jan-2007	−68.17	−0.03			
Recapture	Z-72971	III	8-Feb-2007	−69.82	4.78	16	600	5.34×10^−20^
**Capture**	Z-72930	III	5-Feb-2007	−69.08	8.33			
Recapture	Z-72945	III	6-Feb-2007	−69.37	6.23	12	100	1.25×10^−13^
Recapture	Z-72946	III	6-Feb-2007	−69.37	6.23	12		1.25×10^−13^
**Capture**	Z-72941	III	6-Feb-2007	−69.32	7.22			
Recapture	Z-72935	III	5-Feb-2007	−69.08	8.33	16	45	3.06×10^−17^
	Z-72955	III	7-Feb-2007	−69.60	5.83	16	110	3.06×10^−17^
**Capture**	Z-72944	III	6-Feb-2007	−69.37	6.23			
Recapture	Z-72963	III	8-Feb-2007	−69.67	4.88	16	60	2.48×10^−20^
	Z-72970	III	8-Feb-2007	−69.82	4.78	16	75	2.48×10^−20^
**Capture**	Z-72949	III	7-Feb-2007	−69.40	5.15			
Recapture	Z-72973	III	8-Feb-2007	−69.82	4.78	16	35	2.62×10^−18^

Under Capture Occasion, the first sampling event is listed as the capture and the subsequent sampling events as recapture events. The lab ID code, dates of capture and re-capture(s), with latitude and longitude (lat and long) are reported for each sampling event. For each recapture, the number of matching loci, the minimum distance between locations and the probability of identity (p(ID), as calculated in CERVUS v3) are listed. The two between-year recaptures are listed at the top of the table.

### Haplotype identity and phylogenetic relationships

The control region sequences of the 166 individuals in the IDCR/SOWER samples were aligned with sequences of the 17 individuals from the JARPA samples [15] to give a final circumpolar dataset of 183 individuals. Among the final dataset of 183 individuals, we identified 72 females and 85 males (n = 157). This did not differ significantly from a 1∶1 sex ratio (binomial exact test, p = 0.33).

After trimming to the 410 bp length available for the JARPA samples, we resolved 52 unique haplotypes defined by 47 variable sites: 46 transitions and three transversions (Table S1). A search of GenBank (dated August 15, 2011) showed that 23 of the 52 haplotypes had not been reported previously in any population of blue whales. A total of 11 haplotypes were present in only a single individual (singleton). One variable site outside the 410 bp consensus region resolved one additional haplotype (Z-62480, Table S1), but, for comparative purposes, this was not included in subsequent analyses. All new haplotypes were submitted to GenBank with associated laboratory identification codes (Table 4).

**Table 4:** Frequencies of mtDNA haplotypes for individual Antarctic blue whales spanning IWC management Areas I–VI (n = 184) and genetic sex information if available (f = female, m = male).

Lab ID code (GenBank #)	I	II	III	IV	V	VI	Total	f	m
Z-13951_OSU (JN801048)			4	1			5	1	2
Z-51460_OSU (JN801049)					1		1		1
Z-51461_OSU (JN801050)			9		1		10	6	3
Z-51470_OSU (JN801051)					1		1		1
Z-51472_OSU (JN801052)					1		1	1	
Z-51480_OSU (JN801053)			1		1		2	1	1
Z-51481_OSU (JN801054)			2		2		4	2	1
Z-51488_OSU				1	2		3	1	2
Z-51486_OSU (JN801055)			1				1	1	
Z-62481_OSU (JN801056)			2				2	1	1
Z-62482_OSU (JN801057)			3				3	1	1
Z-62487_OSU (JN801058)			1				1		1
Z-72906_OSU (JN801070)			1				1	1	
Z-72910_OSU (JN801059)			2				2		2
Z-72912_OSU (JN801060)			1				1	1	
Z-72916_OSU (JN801061)			1				1	1	
Z-72917_OSU (JN801062)			2	1			3	1	2
Z-72929_OSU (JN801063)			2				2	1	1
Z-72931_OSU (JN801064)			1				1		1
Z-72935_OSU (JN801065)			1	1			2	1	1
Z-72943_OSU (JN801066)			1				1	1	
Z-72949_OSU (JN801067)			1				1		1
Z-72956_OSU (JN801068)			10		2*		12*	4	3
Z-88257_OSU (JN801069)				1	1		2		2
Z-72930_AA			4				4	1	2
Z-72896_B			2				2	1	
Z-26580_BB				2	6	3	11	6	5
Z-62506_D			4				4	1	3
Hap_EE*				1			1	1	
Z-72907_F			1				1		1
Z-13944_FF			2	1			3		2
Z-13945_GG			3	1			4	1	2
Z-62475_H		1	1				2		2
Z-13948_HH			7	1			8	2	5
Z-62489_I		3	6	1	1		11	1	9
Z-7341_J		2					2		2
Z-7619_K	1						1		
Z-88260_L		1	10	1	1		13	7	5
Z-7622_M			7	1	5	1	14	8	3
Z-13949_N			1	5	1		7	3	3
Z-26574_NN						2	2	1	
Z-26578_OO						2	2	1	1
Z-11165_R		1	1				2	1	
Z-26589_RR	1				2	1	4	3	1
Z-26590_SS				1		1	2		1
Z-26594_TT	1		1				2		1
Z-11164_U		1	2		3		6	1	4
Z-Z51475_UU					2		2		2
Z-26586_V		1				1	2	2	
Hap_X*					1		1	1	
Z-51451_Y			1		4		5	3	2
Z-26591_Z	1		1		1		3	1	2
Total	4	10	101	20	38*	11	184	72	85

Asterisks (*) denote haplotypes found in JARPA samples included from LeDuc et al. [15].

**Figure 2:**
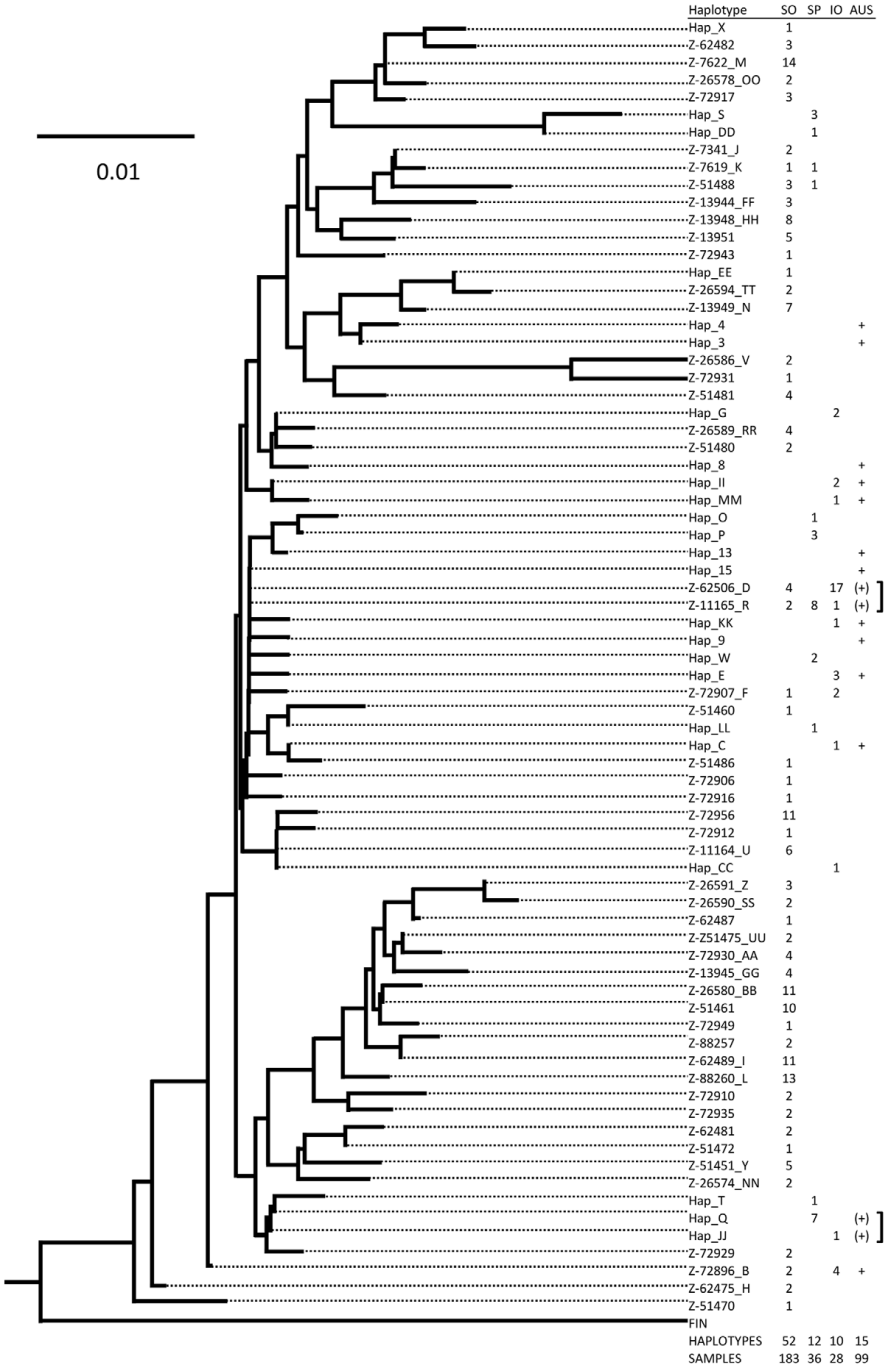
A phylogenetic reconstruction of mtDNA control region haplotypes of Southern Hemisphere blue whale populations. Frequencies of haplotypes are shown according to population or geographic region (SO = Southern Ocean, SP = South-east Pacific Ocean, IO = Indian Ocean, AU = Australia) are listed in the table at the right where available. Previously undescribed haplotypes are listed with the sample lab ID code. Sequences from GenBank are listed as Hap_XX where numeric values refer to haplotypes described by Attard et al. [45] and letter values refer to haplotypes described by LeDuc et al. [15]. Brackets denote where two haplotypes (AU) were not differentiated based on the 396 bp sequence.

To verify the subspecies identity of our sample, we compared the 52 haplotypes from the Antarctic blue whales to other published haplotypes from blue whales in the Southern Hemisphere including the South-east Pacific (n = 10), Indian Ocean (n = 12) and Australia (n = 15) (Figure 2). Neighbor-joining and Bayesian trees were constructed in PAUP* and BEAST, using K2P, K3P and a TIM model for evolution in PAUP* and priors selected by Mr.Modeltest in BEAST. Neighbor-joining and Bayesian phylogenetic reconstructions were similar to that reported by LeDuc et al. [15] in showing only a small number of shared haplotypes (n = 6) between the Antarctic blue whale population and the other Southern Hemisphere populations, but no phylogenetic support for the two subspecies (Figure 2).

We repeated the test of differentation reported in LeDuc et al. [15], using our expanded sample of Antarctic blue whales and the reported frequencies of haplotypes in the South-east Pacific (n = 28) and Indian Ocean (n = 36) populations. Despite the absence of a phylogenetic distinction between Antarctic and ‘non-Antarctic’ blue whales, the three Southern Hemisphere populations were strongly differentiated based on haplotype frequencies and molecular distance (F_st_ = 0.106, p<0.001, Φ_st_ = 0.227, p<0.001; Table 5).

**Table 5:** Pairwise differentiation (F_ST_ italicized below and φ_ST_ above) of mtDNA control region sequences among three populations of blue whales in the Southern Hemisphere: the Antarctic blue whale of the Southern Ocean, as presented in this study, and the pygmy blue whales of the Indian Ocean and South-east Pacific, as reported by LeDuc et al. [15].

	SO (n = 183)	IO (n = 36)	SP (n = 28)
SO		**0.272**	**0.158**
		**p<0.001**	**p<0.001**
IO	***0.113***		**0.209**
	**p<0.001**		**p<0.001**
SP	***0.082***	***0.186***	
	**p<0.001**	**p<0.001**	

Sample sizes are listed for each region. Significance values were based on a permutation test in Arlequin.

### Haplotype diversity and haplotype richness

Based on the final circumpolar dataset of 183 individuals, mtDNA haplotype diversity was 0.968±0.004, with a nucleotide diversity of 1.40±0.70%. Both Tajima's D and Fu's F test were negative, suggesting an excess of rare haplotypes, rather than a recent bottleneck. Only Fu's F test was significant (Fu's F = −24.91, p<0.001,Tajima's D = −0.870, p = 0.197). Using the frequencies of the 52 haplotypes in the circumpolar sample, the rarefaction analysis with the Clench function provided a median estimate of 72 (95% CL, 64–86) for the number of surviving haplotypes in the contemporary population of Antarctic blue whales.

### Geographic differentiation

Based on frequencies of mtDNA haplotypes from the 184 individual samples (i.e., including one individual sampled in 2 Areas), an AMOVA showed significant overall differentiation among the six management Areas (F_ST_ = 0.032, p<0.001; φ = 0.023, p = 0.012). Differentiation remained significant for both males and females when considered separately (males, n = 85 F_ST_ = 0.018, p = 0.009; females n = 73, F_ST_ = 0.044, p<0.001). Pairwise values for F_ST_ between Areas ranged from 0.028 to 0.082 (Table 6). Excluding Area I, which was considered too small for statistical analysis, seven of the ten pairwise comparisons were significant at p<0.05. Most of the non-significant comparisons also involved Areas with small sample sizes.

**Table 6:** Pairwise differentiation (F_ST_ italicized below and φ_ST_ above) of Antarctic blue whale mtDNA haplotypes in IWC management Areas I–VI.

Area	I (n = 4)	II (n = 10)	III (n = 101)	IV (n = 20)	V (n = 38)	VI (n = 11)
I		0.020	0.080	0.000	0.031	0.012
permutation		0.358	0.088	0.620	0.273	0.388
exact test		-------	-------	-------	-------	-------
II	*0.052*		0.000	0.039	0.002	0.024
	0.178		0.561	0.136	0.388	0.301
	0.170		-------	-------	-------	-------
III	*0.023*	***0.034***		**0.051**	0.004	0.044
	0.204	**0.035**		**0.004**	0.240	0.050
	0.012	0.060		-------	-------	-------
IV	*0.036*	***0.053***	***0.028***		**0.037**	0.056
	0.263	**0.027**	**0.006**		**0.041**	0.066
	0.235	0.055	**0.005**		-------	-------
V	*0.014*	***0.052***	***0.027***	*0.021*		0.005
	0.327	**0.011**	**0.000**	0.052		0.331
	0.158	**0.012**	0.000*	0.059		-------
VI	*0.032*	***0.082***	***0.059***	*0.039*	*0.012*	
	0.294	**0.011**	**0.002**	0.051	0.231	
	0.195	**0.015**	0.000*	0.152	0.230	

Sample sizes are listed for each IWC management Area and p-values are listed under the φ_ST_ or F_ST_ values. Upper p-values were based on 5,000 permutations of the data matrix and lower p-values were based on an exact test of differentiation (for F_ST_ only) as implemented in Arlequin. F_ST_ and p-values are bolded if significant at p<0.05. Significant pairwise comparisons after a sequential Bonferroni correction are noted with an asterisk (*). Area I is reported for clarity but sample size was not considered sufficient for statistical tests.

Although the primary objectives of our study were to characterize diversity and differentiation of mtDNA, we also investigated differentiation in microsatellite allele frequencies among the six management Areas. For this we used the genotypes from the 163 individuals for which DNA was available (i.e., we did not include the 17 JARPA samples or the three IDCR/SOWER samples that did not amplify for mtDNA). Three loci showed evidence of deviation from Hardy-Weinberg Equilibrium after a sequential Bonferonni correction, (GATA417, Ev104, Ev21). Of these, GATA417 and Ev104 showed an excess of homozygotes, consistent with a Wahlund effect due to population structure (see below). The third locus, Ev21, showed an excess of heterozygotes but removal of this locus did not affect results of the tests of differentiation and it was retained in this analysis for consistency with the genotype identification (Table 2). Given the variability in success of genotyping at all 16 loci and thus some variability in the sample sizes for each Area, we also repeated the analysis using a subset of seven loci for which >80% of samples were genotyped (Table 2). For both analyses (e.g., 16 loci and 7 loci) we found weak but significant overall differentiation (16 loci, F_ST_ = 0.005, p = 0.031; 7 loci, F_ST_ = 0.010, p = 0.031). At 16 loci, only one pairwise comparison was significant, Area III and V (F_ST_ = 0.007; p = 0.010). At seven loci, two pairwse comparisons were significant: Area III and V (F_ST_ = 0.012, p = 0.003), and Area III and VI (F_ST_ = 0.033, p = 0.042).

Using the seven loci with the most complete coverage of individuals, STRUCTURE did not find any evidence of cryptic population structure. Although the Δk method of Evanno et al. [43] supported k = 2, examination of individual assignment probabilities were close to 0.5. This suggested no detection of true population structure, given that individuals were being assigned at random to k populations.

## Discussion

### Individual movement

The genotype identification of individuals sampled in the IDCR/SOWER cruises provided the first evidence of large-scale movements of Antarctic blue whales since the end of the ‘*Discovery*’ mark program nearly 50 years ago. We identified two between-year recapture events, one of which documented the movement of a female between Area III and Area V, a minimum distance of approximately 6,650 kilometers. The other between-year recapture and the eight within-year recaptures occurred within the same Area (III). Although the number of recapture events was small, the trends of longitudinal movement and time elapsed between re-sampling events are consistent with those from the ‘*Discovery*’ marks [9]. The majority of longitudinal movement as inferred from the ‘*Discovery*’ marks remained within 60° longitude of their implantation location (i.e., within the longitudinal span of a management Area) but over an elapsed time of several years, some individuals showed movement of up to 180° longitude. These trends are also comparable to those from preliminary analyses of photo-identification records collected during IDCR/SOWER cruises in showing a tendency for seasonal residency and annual return within Areas, despite some movement between Areas [44].

### Antarctic and ‘non-Antarctic’ blue whales

Our comparison of mtDNA haplotypes from blue whales in the Southern Hemisphere was consistent with that of LeDuc et al. [15] in showing strong differentiation between the Antarctic blue whale and other ‘non-Antarctic’ populations (e.g., Indian Ocean and South-east Pacific) but no evidence of phylogenetic distinctiveness between the two putative subspecies. For the purposes of understanding the impact of exploitation on mtDNA diversity and differentiation, however, we considered it sufficient to confirm the autonomy of the Antarctic blue whale as a genetic management unit, not to resolve the complex taxonomy of the blue whale. Although we detected a small number of shared haplotypes (n = 6) between the Antarctic and ‘non-Antarctic’ blue whales, it was not possible to judge whether these were the result of misclassifications (i.e., ‘vagrant’ in LeDuc et al. [15]) or incomplete lineage sorting in the recent evolutionary history of these populations. While the inclusion of a small number of ‘non-Antarctic’ haplotypes could inflate our estimates of diversity and differentiation, we considered this preferable to a downward bias that would result from falsely excluding true Antarctic haplotypes. Standardization of nuclear markers for assignment procedures, a larger sample of ‘non-Antarctic’ populations (e.g., Attard et al. [45]) or the use of mitogenomics [46] could better inform future classification of samples.

### Loss of haplotype diversity and haplotype richness?

The Antarctic blue whale population has retained high levels of mtDNA haplotype diversity, despite an estimated decline to less than 1% of pre-exploitation abundance [5]. Haplotype diversity within the Antarctic blue whale population was 0.968, slightly lower than the previously reported value of 0.987 by LeDuc et al. [15] but higher than other ‘non-Antarctic’ blue whale populations in the South-east Pacific, and Indian Ocean (Table 7). Like the humpback whale, which has also retained relatively high haplotype diversity in the Southern Hemisphere, the blue whale may have escaped a marked loss in haplotype diversity due to its longevity, overlapping generations and the relatively brief duration of the population bottleneck [47]. The minimum size of the Antarctic blue whale population (i.e., the ‘exploitation bottleneck’) is estimated to have occurred in 1972 [4], [48], after which there were no further reported legal or illegal catches. This bottleneck is less than 20 years before the initiation of sample collections by IDCR/SOWER in 1990. Given the longevity of Antarctic blue whales (probably greater than 65 years; [49]) and subsequently long generation time (31 years; [50]), individuals that lived through the bottleneck are likely to be alive today. Even allowing for one or two generations of drift in a population with a minimum census size of about 400, the predicted loss of 1–2% in haplotype diversity would be hard to detect in the IDCR/SOWER samples [51].

**Table 7:** Species, regional population, sequenced base pair length (bp), sample size (n), number of haplotypes (ĥ), haplotype diversity (*h*) and nucleotide diversity (π) for mtDNA haplotypes for several species of great baleen whales in comparison with blue whales.

Species\subspecies	Regional population	bp	n	ĥ	*h* (SD)	π (%) (SD)	Reference
**Blue whale**							
*B. musculus*							
*B. m. brevicauda*	Indian Ocean	414	36	12	0.765 (0.070)	n.a.	[15]
*B. m. brevicauda*	South-east Pacific Ocean	414	28	10	0.852 (0.042)	n.a.	[15]
*B. m. brevicauda*	Bonney Upwelling	396	32	9	0.758 (0.070)	0.40 (0.30)	[45]
	Perth Canyon	396	67	14	0.683 (0.062)	0.30 (0.20)	[45]
*B. m. intermedia*	Southern Ocean	414	47	26	0.987 (0.010)	n.a,	[15]
	Southern Ocean	410	183	52	0.968 (0.004)	1.61 (0.86)	this study
**Southern Right Whale**							
*E. australis*	South Atlantic basin	275	69	28	0.948 (0.013)	2.90 (1.51)	[10]
	Indo-Pacific basin	275	67	7	0.701 (0.037)	2.03 (1.09)	[10]
**North Atlantic Right Whale**	Western North Atlantic	500	269	5	0.698 (0.016)	0.60 (0.30)	[69]
*E. glacialis*							[70]
**North Pacific Right Whale**	North Pacific	540	5	2	0.600 (0.129)	1.89 (1.22)	[70]
*E. japonica*							[71]
**Gray Whale**							
*E. robustus*	western Pacific	523	45	10	0.70 (0.05)	1.7 (n.a.)	[72]
	eastern Pacific	523	120	33	0.95 (0.01)	1.6 (n.a.)	[72]
**Humpback Whale**							
*M. novaeangliae*	South Pacific	470	1,112	115	0.975 (0.001)	2.04 (1.03)	[11]
	Worldwide	283	90	37	0.88	2.57 (n.a.)	[47]
**Minke Whale**							
*B. bonaerensis*	Brazil	500	61	47	n.a.	1.6 (0.1)	[73]
	Antarctic	500	119	83	n.a.	1.5 (0.1)	[73]
*B. acutorostrata*	eastern North Pacific	500	6	5	n.a.	0.6 (0.2)	[73]
	western North Pacific	500	127	34	n.a.	1.0 (0.5)	[73]
	Sea of Japan	500	28	3	n.a.	0.6 (0.1)	[73]
	Brazil	500	8	3	n.a.	1.2 (0.6)	[73]
	Antarctic	500	15	8	n.a.	0.7 (0.1)	[73]
**Bowhead whale**							
*B. mysticetus*	Bering-Chukchi-Beaufort Seas	493	98	68	0.986 (0.005)	1.63 (0.09)	[74]

However, the most sensitive measure of a bottleneck is likely to be loss of haplotype richness, rather than haplotype diversity [52], [53]. Here, it seems our minimum census of 52 haplotypes from 183 individual Antarctic blue whales is low compared to the 68 haplotypes reported in 98 bowhead whales (*Balaena mysticetus*) or the 83 reported in 119 Antarctic minke whales (*Balaenoptera bonaerensis*) (Table 7). As the Antarctic minke whale represents the only abundant species that was little depleted by whaling, it represents perhaps the best proxy of ‘pre-exploitation’ genetic diversity [54]. Unfortunately, comparisons of haplotype richness are highly dependent on standardization of both the length of the sequence and sample size. For this, our estimate of 72 surviving haplotypes in the Antarctic blue whale is most comparable to the estimate of 68 for southern right whales, where both were standardized for sequence length and for sample size using a rarefaction analysis [18]. Although both species were intensively exploited, the similarity in surviving haplotypes is somewhat surprising given that southern right whales were depleted in the early 19^th^ century, reaching an estimated minimum population size of about 300 in 1920 [18]. This earlier exploitation would have allowed another several generations of drift and presumably greater loss of haplotypes than for the more recently exploited Antarctic blue whale. However, it is possible that the circumpolar loss of haplotypes in southern right whales has been mitigated by the stronger subdivision of this species into discrete breeding or calving grounds [10].

### N_min_ and historical population dynamics

The number of observed or estimated mtDNA haplotypes in contemporary populations of exploited whales can provide an absolute lower boundary on the number of females to survive the exploitation bottleneck [55]. This lower boundary can be used to inform or constrain population dynamic models used by the IWC to reconstruct the historical trajectory of decline and recovery (if any) of exploited whales [18]. In the absence of this lower boundary, population trajectories reconstructed from density-dependent models are often compatible with relatively high rates of intrinsic increase and very low abundance at the time of the exploitation bottleneck (referred to as *N_min_*). Setting a lower boundary on *N_min_* can exclude high rates of intrinsic increase, which, in turn, can increase the pre-exploitation abundance estimated from population dynamic models. The effect of constraining *N_min_* is more marked for populations such as the Antarctic blue whale, which have undergone a very narrow population bottleneck, as density-dependent models assume that rates of increase will be near maximum at this low point in the historical population trajectory.

A recent attempt by the IWC to reconstruct the pre-exploitation abundance and decline of the Antarctic blue whale used an *N_min_* of 214 to constrain the lower boundary of historical population trajectories [48] as summarized in [4]. This value was derived, after various adjustments [17], from the 26 haplotypes reported in LeDuc et al. [15]. Our minimum census of 52 haplotypes has doubled this count and our prediction of 72 surviving haplotypes exceeds the previous prediction of 51, estimated by the same rarefaction approach [17]. On the other hand, Branch and Jackson [17] assumes that the number of haplotypes would increase by 29% with longer sequence lengths, whereas this study differentiated only one additional haplotype with longer sequences. The combined effect of these changes could increase estimates of *N_min_*, which would further constrain the range of historical reconstructions predicted from the population dynamic model. If so, this is likely to increase the estimate of pre-exploitation abundance, but decrease the estimate for rate of increase [4], [48]. However, the magnitude of these changes cannot be evaluated without reimplementation of the full model.

### Population structure and maternal fidelity

Despite the absence of physical barriers and the unlimited mobility of Antarctic blue whales, we found evidence of significant population structure based on the *a priori* boundaries of the management Areas. These divisions were established based on the distribution of catches during the early 20^th^ century, but their biological importance has remained in question. We found stronger population structure for the maternally inherited mtDNA (F_ST_ = 0.032, p<0.001) than for the biparentally inherited microsatellites (F_ST_ = 0.005, p = 0.031), suggesting that this population structure is likely to be the result of maternal fidelity to feeding grounds, similar to that reported previously in some other species of whales (i.e., fin whales in the North Atlantic [56], humpbacks in the North Pacific [57], [58] and North Atlantic [59], [60]). Whether this fidelity relates only to feeding grounds or extends to some concordance with unknown breeding grounds remains unknown. Further, we acknowledge that the management Areas are only a proxy for the oceanographic features likely to be influencing the distribution and population structure of blue whales within the Southern Ocean. However, we were limited in our ability to explore more complex seascape scenarios or to benefit from non-*a priori* clustering methods by the small number of samples in some Areas and the relatively weak levels of differentiation in the molecular markers.

### Genetic monitoring of Antarctic blue whales

Given the longevity and recent history of exploitation, loss of mtDNA diversity in the Antarctic blue whale is probably still ongoing, despite the increase in abundance reported from the IDCR/SOWER sighting surveys [5]. The majority of the 52 Antarctic blue whale haplotypes are present in the population at low frequencies and 15 were represented only by males. These haplotypes will be lost with the eventual death of these males, unless they are shared with related females not included in our sample. Looking forwards, genetic monitoring of Antarctic blue whales over the next several decades would allow a direct measurement of loss in haplotype and allelic richness across the lifespan of whales that survived exploitation [61]. Looking backwards, a direct measurement of the loss of genetic diversity could be derived from samples collected during the early decades of the 20^th^ century, prior to the most intensive periods of whaling. Museum collections [62] and bones or artifact from early whaling stations [63], could provide an invaluable archive for reconstructing the history of intense exploitation and the narrow survival of the Antarctic blue whale.

## Supporting Information

Table S1 (XLSX)
